# Progress of Surgery

**Published:** 1874-03

**Authors:** Jno. E. Owens

**Affiliations:** Lecturer on Surgery, Rush Medical College, Chicago


ARTICLE II.—*Progress of Surgery*. By JNO. E. OWENS, M.D., Lecturer on Surgery, Rush Medical College, Chicago.

1. *The Treatment of Joint Affections by Massage.* Arranged by W. R. FISHER, M.D., after oral translations from the Danish by GEO. R. CUTTER, M.D. (*Medical Record*, New York, Jan. 1, 1874.)

2. *A Successful Case of Abdominal Section for Intussusception.* With remarks on this and other methods of treatment. By JONATHAN HUTCHINSON, F.R.C.S. (*The Medical Press and Circular*, London, Nov. 26, 1873.)

3. *Remarks on Certain Recent Papers on the Action of Alcohol.** By DR. ANSTIE. (*The Practitioner*, London, December, 1873.)

1. We make the following extract from Dr. Fisher's paper:

The word massage (Gr. μάσσω: to knead) has been adopted by the Danish writers from the French, and signifies the use of such passive manipulations as rubbing, kneading, percussing, and rolling of the soft parts. Some of the authors extend its meaning so as to include passive movements of the joints; while others restrict it to manipulations which can be given with the fingers, and use the term effleurage to designate hand-rubbing. The reader may understand the word massage, whenever it occurs in this paper, to mean passive manipulations with the fingers or hand; when movements of the joints are spoken of, the term passive movement will be employed. The attention of the profession in Denmark has been especially directed towards the subject of massage by the widespreading reputation of Dr. Mezger, a Dutch physician now

* This paper is arranged under the heading Progress of Surgery, in consequence of the bearing which such experiments have upon the treatment of pyæmia, septicæmia and erysipelas.—O.

practicing at Bonn, who has recently achieved an increased celebrity by a successful employment of this means in the treatment of the Danish Crown-Prince. The papers (of which Dr. Fisher's article is a translation) all relate to Mezger's method, as it is called; and since the reputation of this physician is deeply dependent upon his success in the treatment of joint affections, although he extends his method to a variety of other complaints, this abstract is confined to the consideration of that class of diseases. It should be borne in mind that the American method of treating joint inflammations by extension and counter-extension, without preventing locomotion, is rarely used anywhere in Europe, and in Denmark and the Scandinavian countries has probably scarcely been heard of; so that the objections which are hereafter, in the course of this paper, to be made against "the usual methods of treatment," should be understood to refer, for the most part, to the use of counter-irritants and the plaster of Paris bandage.

Dr. Mezger employs massage in treating both acute and chronic synovitis in the articulations of the extremities. In the case of the hip-joint, however, he does not use it; partly on account of the deep situation and anatomical relations of this articulation, and partly from the fact that its inflammation often depends upon primary osteitis. He divides his frictions into two classes: the first, passing from side to side, (horizontal); the second, passing from below upwards in the line of the limb, (vertical friction). The applications vary in force according to the effect which he desires to produce, and are made, not only upon the joint itself, but also upon the adjacent unaffected tissues. By means of the horizontal frictions the skin is moved about over the fasciæ and ligaments, and the superficial vessels are acted upon, partly by the direct application of mechanical force, and partly by the indirect influence of the vaso-motor nerves. The circulation of the blood is thereby increased; and where there is a tendency to venous stagnation, the bluish color is removed, and the skin assumes its natural appearance. The vertical frictions are made in the direction of the circulation of fluid in the venous and lymphatic vessels, and promote the flow within them. By a combination of these two methods of manipulation—the one stimulating the action, and the other propelling the contents of the blood vessels and lymphatics—absorption is necessarily increased. Still farther, where there are deposits or effusions of blood in the soft parts, the use of frictions may bring about slight changes in these portions, and tend to disperse them, by breaking them up and rendering them more readily absorbable. Massage, therefore, is a powerful agent in producing the absorption of effused materials; acting both by preparing them for removal, and also by promoting the proper functions of the lymphatic vessels. Compression will likewise promote absorption, by the lymphate, but it acts upon the subcutaneous veins, and gives rise to œdema of the parts below the points of application; whereas by the use of massage, not only is there no obstruction offered to the venous flow, but, on the contrary, the circulation is greatly promoted. For these reasons massage can be used with great confidence in the treatment of synovitis. In acute and chronic synovitis, the vertical frictions are more applicable than the horizontal. According to Mezger, the average time which is required for the treatment of an acute synovitis by this method is two weeks, and for a chronic case six weeks. The hyperplastic forms of synovitis require a somewhat more complicated treatment. (All cases which have advanced to suppuration are excluded.) Both the horizontal and vertical frictions are to be employed, and usually strong pressure is required, especially over those points where the peri-synovial tissue is felt to be much thickened. The more acute the inflammatory process the softer should be the pressure, as a general rule. Synovitis-hyperplastica presents a hyperplasia of the anolar tissue in the synovial membrane and the peri-synovial tissues, together with a more or less plentiful serous exudation. At the same time there is a development of newly formed vessels. Usually a more protracted course of treatment becomes necessary. Dr. Mezger recommends to his patients, both in acute and chronic cases, a moderate use of the affected joint, to an extent which is limited by the production of pain; passive movements are also used by him at the same time. Dr. Mezger's chief merit consists in the fact that he has separated massage from the system of therapeutic gymnastics of which it forms a part, and that he has studied its effects upon diseases more thoroughly and completely than has ever been or could have been done while it remained in the position of accessory to other treatment. Mezger has elevated massage to the dignity of chief therapeutic means in his treatment of joint affections, and passive movements assume with him a secondary importance.

At a meeting of the Medical Society of Copenhagen, September, 1872, Dr. P. Winge made some practical remarks upon Mezger's method. He describes it briefly as consisting essentially in kneading, rolling, percussing, and rubbing the parts which are the subject of treatment. The operator sits in front of his patient on a low stool, and begins by oiling the part with perfumed lard. He rubs strongly whenever indurations, infiltrations or effusions are to be dealt with, and follows from below upwards the course of the lymphatic vessels in the extremities. When the knee-joint is the subject of treatment, he works across the joint with the fingers of one hand, on both sides, below the patella, pressing inwards with more or less force; while the fingers of the other hand work in the same manner upwards along both sides of the patella, over the capsular ligament, or any ligament which is felt to be swollen. This process is continued from three to five minutes. He then grasps the joint with the right hand, pressing firmly, rubs upwards over the patella, as high as the superior insertion of the investing ligaments. The applications are repeated once or twice every day.

2. The patient was a child aged two years. The intussusception had commenced at the cæcum, and was of such length that its extremity, presenting the inverted ilio-cæcal valve, was extruded several inches at the child's anus. The condition had been one month in the course of development; latterly the case had been treated as one of prolapsus, and attempts had been made to keep the bowel in place by means of a cork pad. The child was very ill, and the author having failed in attempts to effect reduction by enemata, etc., and having had experience of several similar cases which had ended fatally, determined to operate. The child was put under chloroform, and the abdomen was opened in the middle line below the umbilicus. The intussusception was then easily found, and as easily reduced. The after treatment consisted only in the administration of a few mild opiates, and the child made a rapid recovery. The author next narrated briefly the particulars of those somewhat similar cases in which he had been consulted, and in which the intussuscepted bowel could be easily felt by the finger in the rectum. In all these, in spite of persevering treatment by injections, bougies, etc., the patients had died unrelieved. From a consideration of cases, the following conclusions were suggested:

1. That it is by no means very uncommon for intussusception to begin at the ilio-cæcal valve, and to progress to such a length that the invaginated part is within reach from the anal orifice, or even extruded.

2. That it is of great importance in all suspected intussusception to examine carefully by the anus.

3. That in almost all cases of intussusception in children, and probably in most of those in adults, the diagnosis may be made certain by handling the invaginated part through the abdominal wall.

4. That in a large proportion of the cases in which children under one year are the patients, death must be expected in from one to four or six days from the commencement.

5. That in fatal cases death is usually caused by shock, or by collapse from irritation, and not by peritonitis.

6. That in many cases it is easy, by estimating the severity of the symptoms (vomiting, constipation, etc.), to form an opinion as to whether the intestine is strangulated, or simply irreducible.

7. That in cases of strangulated intussusception, whilst there is great risk of speedy death, there is also some hope that gangrene may be produced, and spontaneous cure result.

8. That in cases in which the intussuscepted part is incarcerated and not strangulated, there is very little hope of the occurrence of gangrene, and it is probable that the patient will, after some weeks or months, die, worn out by irritation and pain.

9. That the chances of successful treatment, whether by the use of bougies, or by the injection of air or water, are exceedingly small, excepting in quite recent cases, and that if the surgeon does not succeed by them promptly, it is not likely that he will succeed at all.

10. That the cases best suited for operation are those which have persisted for some considerable time, and in which the intestine is only incarcerated; and that these cases are also precisely those least likely to be relieved by any other method.

11. That in cases just referred to, after failing by injections, bougies, etc., an operation is to be strongly recommended.

12. That the records of post mortems justify the belief that in a considerable number of the cases referred to, the surgeon will encounter no material difficulty after opening the abdomen.

13. That the circumstances which might cause difficulty, are—(1) the tightness of the impaction of the parts; (2) the existence of adhesions; and (3) the presence of gangrene.

14. That in selecting cases suitable for operation, the surgeon should be guided by the severity of the symptoms to an estimate of the tightness of the strangulation, and as to the probability of gangrene having already set in.

15. That in cases in which the patient's symptoms are very severe, or the stage greatly advanced, it may be wiser to decline the operation, and trust to the use of opiates.

16. That the operation is best performed by an incision in the median line below the umbilicus.

17. That in cases of intussusception in young infants, under one year of age, the diagnosis is very desperate.

18. That the last fact mentioned may be held to justify, in the case of young infants, very early resort to the operation.

3. The influence of alcohol in reducing temperature is a fact so contrary to preconceived ideas, that until within the last few years it was never even suspected. The attention of Professor Binz was drawn to the subject while he was still unaware of the researches of Ringer and Rickards; and at his instigation M. Curry Bouvier made a series of experiments, in the laboratory at Bonn, with very decided results. On healthy men and on animals, alcohol in moderate doses produced a small, and in large doses a considerable, reduction of heat. Moreover, a dog which had been thrown into a pyrexial state by the injection of pus into the subcutaneous tissue, had its abnormal temperature markedly reduced by considerable doses (each about one drachm absolute alcohol with water); the lowering amounted to more than two degrees in all. The alcohol was then discontinued, the temperature rapidly rose again, and went on rising till death. At a recent meeting of the British Association, Professor Binz, though necessarily in somewhat popular style, laid down, as the definite result of recent experiments, that alcohol always reduces temperature, but that the effect was most readily perceptible in feverish persons, in whom bodily heat was much above the normal line.

I cannot help remarking here (says the author of this paper), on the singular and even grotesque course which English popular medical opinion has taken on this question of alcohol in pyrexia, since the death of Todd. His observations, which were doubtless inexact (for medical thermometry did not in his time exist), have been declaimed against in every variety of tone; but the special reproach against them has always been that they were "not truly scientific." Loud as this outcry has been, I am not aware that (before Ringer and Rickards) a single English medical observer (one or two of Todd's pupils excepted) had ever put to the simple test of the thermometer, Todd's main allegation—that alcohol reduces febrile temperature by its direct action. That very portion of Todd's practice which has excited the fiercest condemnation in this country, viz., his administration of very large doses of alcohol in conditions of high pyrexia, turns out to agree best with the results of physiological investigation, and has in practice been endorsed by such eminent physicians as Liebermeister, Binz, Socin, and many others. The Franco-German war gave abundant occasion for testing this matter; and it was fortunate that Professor Binz received a high medical military post, and that such men as Socin and others, who also filled important medical posts in the war, were willing to carry out his plans with vigor. Those who are acquainted, either personally or through his writings, with Professor Socin (Basle), are aware that a more accomplished observer is not to be found in Europe; it need, therefore, hardly be said that his war experiences of the treatment of pyæmia, erysipelas, etc., possess an uncommonly high value. It is therefore a matter of much importance that we find him stating that in the severe septicæmic wound-fevers he not only employed quinine in enormous daily quantity (20 to 105 grains), but at the same time gave three bottles of wine every day. Under this treatment he saw many unexpected improvements, and even recoveries; and he declares that the wine not only increased the lowering effect of the quinine on the temperature, but that it also much moderated the toxic effects; *i. e.*, the "cinchonine." Erysipelas was also a disease that came very largely under Socin's observations at Carlsruhe, and he speaks here in a different manner. He specially remarks that quinine had but little or no effect in reducing temperature, and all remedies appear to have been unavailing to reduce the temperature very markedly. Nevertheless, the patients supported the high temperature particularly well, and he attributes this, at least in part, to the administration of three to four bottles daily, of champagne and sherry mixed. The researches of Binz have gone far to prove that the cooling influence of alcohol is not exerted through the heat-regulating centres. In his papers in Virchow's Archiv. (1870) will be found the record of experiments in which the possible interference of such a course was elaborately provided against. And the general result of the inquiries is to reinforce more strongly than any other researches have done for a long time, the opinion that alcohol, while itself oxidized within the blood with great rapidity, hinders the oxidation of the tissues. Hence it can be easily understood that the effect of alcohol in reducing temperature would be most plainly seen in the febrile diseases, when the tissues are being violently consumed, while the supplies of ordinary nutriment are necessarily very small. Such, in fact, proves to be the case, and Dr. Binz has recently informed me (last August) that whereas in smaller doses, *e. g.*, one ounce of wine in half an ounce of brandy, it is usually not possible to obtain a reduction of more than a few decimal parts of a degree, and the dose must be frequently renewed to maintain the effect, he has found that the influence of a very large dose has sufficed to produce a reduction amounting to several degrees. Whether this be, on the whole, the best method of reducing pyrexia by means of alcohol, is a question which will be found more fully investigated in my new researches, which will shortly be published.*

* My own experience in the use of very large doses of alcohol, in the treatment of pyæmia and septicæmic fever, leads me to attribute the unexpected improvements and occasional recoveries more to an antidotal power of the agent than to its influence in reducing temperature. The patients die from poisoned blood, and not from high temperature. Can it be that the reduction of the temperature is only a measure of this antidotal quality of the alcohol?—O.

